# Development and validation of an online dynamic prognostic nomogram for incidental gallbladder adenocarcinoma patients without distant metastasis after surgery: a population-based study

**DOI:** 10.3389/fmed.2023.1175211

**Published:** 2023-11-10

**Authors:** Jie Chen, Yehong Han

**Affiliations:** ^1^Department of General Surgery, Hangzhou TCM Hospital Affiliated to Zhejiang Chinese Medical University, Hangzhou, China; ^2^Department of General Surgery, Hangzhou Hospital of Traditional Chinese Medicine, Hangzhou, China

**Keywords:** incidental gallbladder adenocarcinoma, dynamic nomogram, overall survival, cancer-specific survival, SEER

## Abstract

**Background:**

Gallbladder cancer is the most common malignant tumor of the biliary system, most of which is adenocarcinoma. Our study explored developing and validating a nomogram to predict overall and cancer-specific survival probabilities internally and externally for incidental gallbladder adenocarcinoma patients without distant metastasis after surgery.

**Methods:**

Patients screened and filtered in the Surveillance, Epidemiology, and End Results (SEER) database, whose years of diagnosis between 2010 and 2015 were collected as a derivation cohort, while those between 2016 and 2019 were a temporal validation cohort. Overall survival (OS) and cancer-specific survival (CSS) were chosen as the primary and secondary endpoints of the retrospective study cohort. Potential clinical variables were selected for a Cox regression model analysis by performing both-direction stepwise selection to confirm the final variables. The performance of final nomograms was evaluated by Harrell’s C statistic and Brier score, with a graphical receptor operating characteristic (ROC) curve and calibration curve.

**Results:**

Seven variables of age, race, tumor size, histologic grade, T stage, regional lymph nodes removed, and positive regional lymph nodes were finally determined for the OS nomogram; sex had also been added to the CSS nomogram. Novel dynamic nomograms were established to predict the prognosis of incidental gallbladder adenocarcinoma patients without distant metastasis after surgery. The ROC curve demonstrated good accuracy in predicting 1-, 3-, and 5-year OS and CSS in both derivation and validation cohorts. Correspondingly, the calibration curve presented perfect reliability between the death or cancer-specific death probability and observed death or cancer-specific death proportion in both derivation and validation cohorts.

**Conclusion:**

Our study established novel dynamic nomograms based on seven and eight clinical variables separately to predict OS and CSS of incidental gallbladder adenocarcinoma patients without distant metastasis after surgery, which might assist doctors in advising and guiding therapeutic strategies for postoperative gallbladder adenocarcinoma patients in the future.

## Introduction

Gallbladder cancer is the most common malignant tumor of the biliary system, with the tendency of early lymph node metastasis and distant metastasis (1, 2), of which more than 90% are adenocarcinoma (3–5). According to the GLOBOCAN 2020, 115,949 new cases of gallbladder cancer (41,062 male and 74,887 female individuals) and 84,695 deaths (30,265 male and 54,430 female individuals) were reported worldwide, ranking sixth in digestive system tumors, and China is one of the regions with a high incidence (6). Gallbladder cancer confirmed by pathological diagnosis during or after cholecystectomy is considered incidental gallbladder cancer, in which stages T1 and T2 are the most typical (7). Therefore, simple cholecystectomy can achieve long-term survival for patients with Tis and T1a stages (8). In contrast, the T1b stage or above is recommended for re-operation and performing standard or extended radical cholecystectomy according to their stages (8). Fortunately, with the boom of laparoscopy, laparoscopic cholecystectomy (LC) appears to bring about the earlier discovery of gallbladder cancer in some patients, resulting in an increased probability of survival (9, 10).

The American Joint Committee on Cancer (AJCC) Tumor Node Metastasis (TNM) staging system is most commonly applied in clinics for gallbladder cancer (11). However, even if considered in the same TNM stage, the survival probability of gallbladder cancer patients still varies widely, causing poor predicting accuracy. Nomograms are graphical illustrations of clinical prediction models that apply several variables to acquire more accurate and trustworthy diagnostic or prognostic predictions (12, 13). Therefore, they have been considerably adopted in multiple tumors and are reported to be superior to traditional staging systems, such as TNM, for prognostic predictions (14, 15). Similarly, various studies have investigated the role of prognostic nomograms via the SEER database using standard clinical variables for gallbladder cancer (16). Most recently, Lin et al. constructed nomograms that showed better discrimination abilities to predict OS and CSS, assisting in risk stratification to guide gallbladder adenocarcinoma treatment (17). Furthermore, Zhang et al. established a more accurate and effective nomogram to predict the prognosis of patients with non-metastatic gallbladder cancer after surgical resection (18). Moreover, Zhang et al. integrated tumor size with other prognostic factors into a predictive nomogram to predict the CSS of gallbladder cancer patients. Given the above, all these nomogram models utilized the previous version of AJCC TNM classification, instead of the latest updated 8th edition. In addition, either the amounts of samples in these studies were relatively limited, or they did not perform external validation yet.

This study explored developing and validating prognostic nomograms of overall and cancer-specific survival probabilities internally and externally for incidental gallbladder adenocarcinoma patients without distant metastasis after surgery.

## Materials and methods

### Study design and data source

The SEER Program collects cancer incidence data from population-based cancer registries covering approximately 48.0% of the U.S. population. SEER*Stat software (version 8.4.0.1) of the National Cancer Institute was used to collect data. The clinical variables of patients confirmed as gallbladder adenocarcinoma between 2010 and 2019 were retrieved from the SEER database: Incidence—SEER Research Plus Data, 17 Registries, Nov 2021 Sub (2000–2019), using which we undertook a retrospective cohort study. The reference number is 15850-Nov2021. Patients whose years of diagnosis were between 2010 and 2015 were collected as a derivation cohort, while those between 2016 and 2019 were collected as a temporal validation cohort.

The inclusion criteria were as follows: (1) primary site: gallbladder (C23.9), according to the International Classification of Diseases for Oncology, third edition (ICD-O-3); (2) histologic type: adenocarcinoma; (3) only one primary tumor; (4) diagnosis confirmed by positive histology; (5) surgery performed; and (6) the main differences between the 7th and 8th editions of the AJCC TNM staging systems are T2 stage is subdivided as T2a (on the side of the peritoneum) and T2b (on the side of the liver), and N is staged according to the number of positive regional lymph nodes (19). T stage is classified as T1a, T1b, and T2 in the years 2010–2017 and T1a, T1b, T2a, and T2b in the years 2018–2019; complete information on regional lymph nodes removed and positive regional lymph nodes; and M stage is classified as M0; and (7) no radiotherapy or chemotherapy. The exclusion criteria included the following: (1) age < 18 years old; (2) data such as tumor size, histologic grade, and follow-up information missing or incomplete.

### Clinical variables extracted for analysis and transformation

Age, sex, race, tumor size, histologic grade, AJCC T stage, regional lymph nodes removed, positive regional lymph nodes, and specific surgery were all selected for subsequent analysis. According to clinical follow-up outcomes, OS and CSS were chosen as the primary and secondary endpoints. In particular, the X-tile software (version 3.6.1) was used to determine and visualize the best cutoff values of age, tumor size, and regional lymph nodes removed variables in our study (20, 21). In addition, the T2a and T2b stages were consolidated as the T2 stage, and the number of regional lymph nodes was divided into 0, 1–3, and ≥ 4 positive regional lymph nodes.

### Construction and validation of the nomogram for OS and CSS

Statistical analysis was conducted using R software (version 4.1.3). Clinical variables of age, sex, race, tumor size, histologic grade, AJCC T stage, number of regional lymph nodes removed and positive regional lymph nodes, and information on specific surgery were all selected for a Cox regression model analysis (survival package version 3.4-0), in which both-direction stepwise selection by AIC method was performed to confirm the final variables (MASS version 7.3-58.1). Based on the analysis results, we used the rms (version 6.3-0) and nomogramEx (version 3.0) packages to formulate the nomogram for OS and CSS and extracted equations for calculating the OS and CSS probability corresponding to the total points. Furthermore, a web calculator of a dynamic nomogram was built by DynNom (version 5.0.2) and rsconnect (version 0.8.27) packages.

Predictive performance was evaluated by Harrell’s C statistic and Brier score. Then, the established nomogram was subjected to enhanced-bootstrap internal validation (1,000 bootstrap resamples). Concerning the external validation, the linear predictor of each patient in the validation cohort was calculated based on the nomogram, and performance measures of Harrell’s C statistic and Brier score were obtained. Moreover, in both the derivation and validation cohorts, the calibration curve of the nomogram was performed by comparing the predicted 1-, 3-, and 5-year death or cancer-specific death probability with observed death or cancer-specific death proportion (riskRegression package version 2022.03.22). In addition, a ROC curve and the area under the curve (AUC) were calculated to evaluate the accuracy of the nomogram to predict 1-, 3-, and 5-year OS and CSS probability (ggplot2 package version 3.3.6).

## Results

### Characterization of included cases

**Figure 1:**
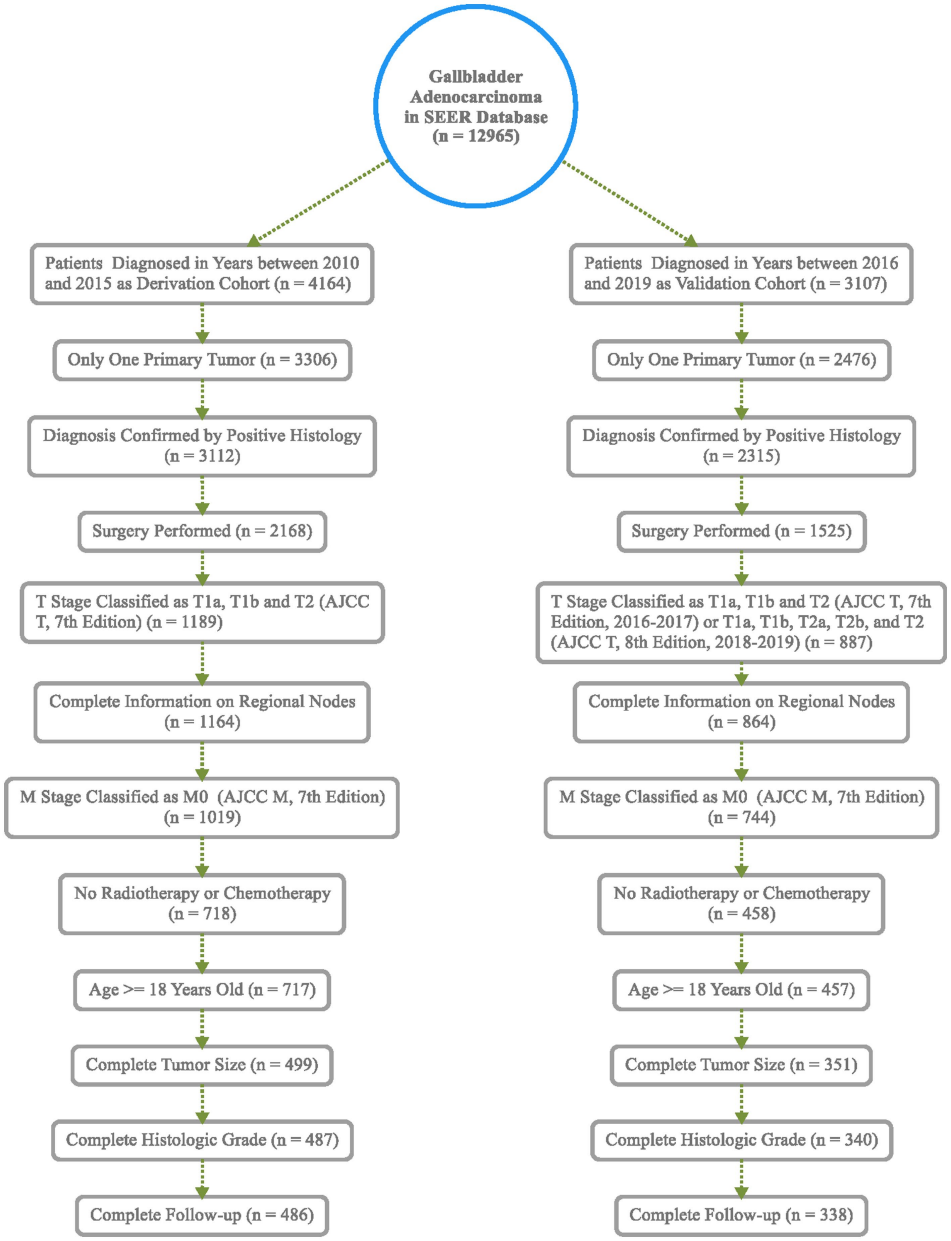
Flowchart of patient selection in the SEER database.

**Figure 2:**
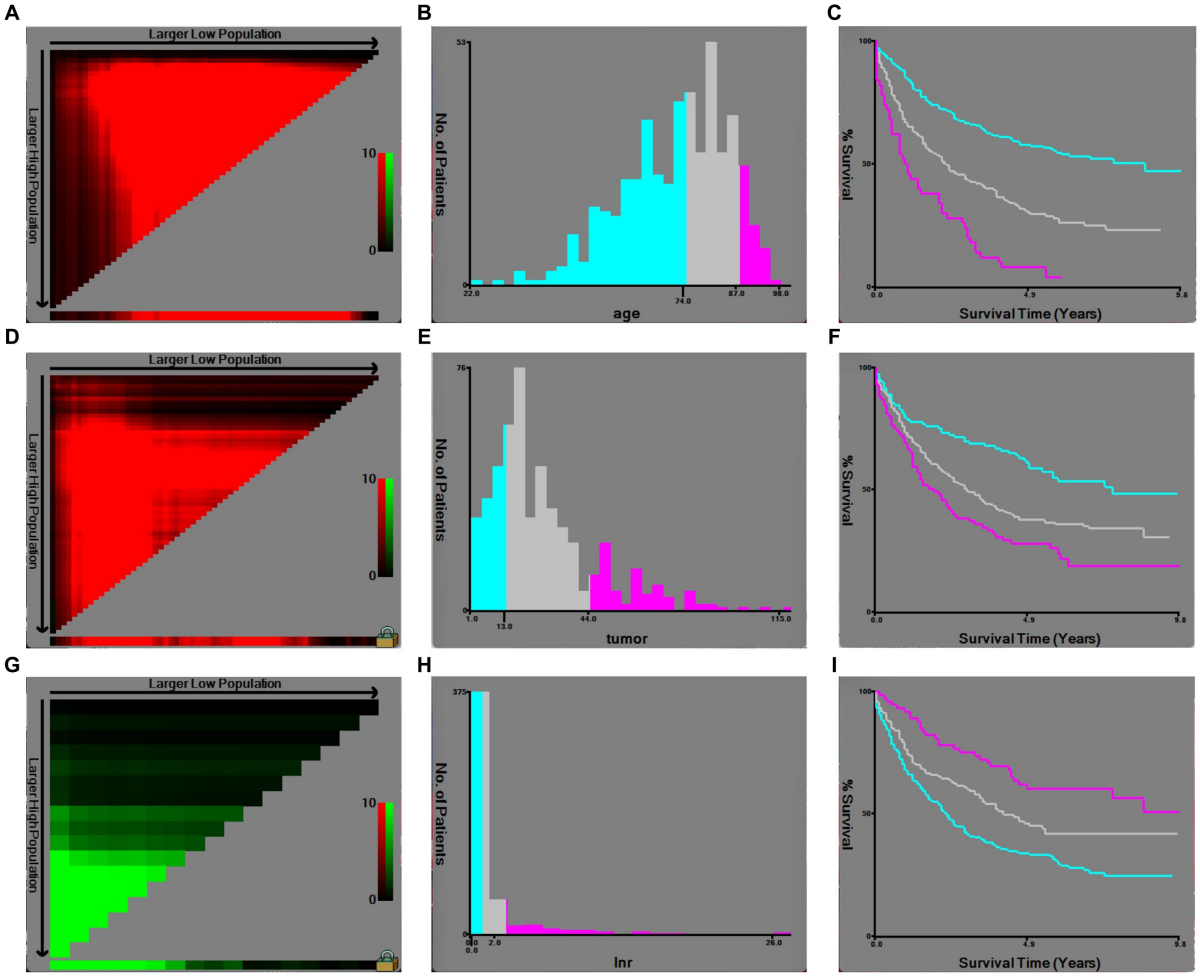
Optimal cutoff values of age, tumor size, and regional lymph nodes removed by X-tile software analysis. **(A)** X-tile plot of age in the derivation cohort; **(B)** optimal cutoff value of age highlighted by a histogram; **(C)** Kaplan–Meier plot of prognosis determined by the optimal cutoff value of age; **(D)** X-tile plot of tumor size in the derivation cohort; **(E)** optimal cutoff value of tumor size highlighted by a histogram; **(F)** Kaplan–Meier plot of prognosis determined by the optimal cutoff value of tumor size; **(G)** X-tile plot of regional lymph nodes removed in the derivation cohort; **(H)** optimal cutoff value of regional lymph nodes removed highlighted by a histogram; **(I)** Kaplan–Meier plot of prognosis determined by the optimal cutoff value of regional lymph nodes removed.

Based on inclusion and exclusion criteria, 486 patients diagnosed between the years 2010 and 2015 were included in the derivation cohort; then, a total of 338 cases diagnosed from 2016 to 2019 were finally selected in the validation cohort (Figure 1). In general, most of the patients were female individuals (71.2% in derivation vs. 68.6% in validation) and white (75.9% in derivation vs. 74.6% in validation). The rates of T1a, T1b, and T2 were 9.5, 17.3, and 73.3% in the derivation population, and 9.2, 15.1, and 75.7% in the validation population, respectively. Overall, the majority of cases were in AJCC stage II (63.6% in derivation vs. 66.0% in validation), a great number of them have no positive regional lymph nodes (90.3% in derivation vs. 88.5% in validation), and the grade of nearly half of the patients (48.4% in derivation vs. 53.3% in validation) was moderately differentiated. Among all patients, the mean and median follow-up for the derivation cohort were 40.3 and 34.0 months and 16.5 and 13.0 months in the validation cohort, respectively. Meanwhile, 196 were cancer-specific deaths, 93 died of other causes in the derivation patients, 69 were cancer-specific deaths, and 24 died of other causes in the validation patients (Table 1). The cutoff points of age, tumor size, and regional lymph nodes removed were decided by X-tile (Figure 2). In particular, 50.6 and 50.9% were ≤74 years old, 39.1 and 37.6% were between 75 and 87 years old, and 10.3 and 11.5% were ≥88 years old separately in derivation and validation groups; 24.3 and 25.4% were ≤13 mm, 58.4 and 58.3% were between 14 and 44 mm, and 17.3 and 16.3% were ≥45 mm separately in derivation and validation groups. Furthermore, most of them have no regional lymph nodes removed (54.9% in derivation vs. 45.9% in validation) (Table 1).

**Table 1:** Demographics and clinical characteristics of patients in the derivation and validation cohorts.

	Derivation cohort (*N* = 486)	Validation cohort (*N* = 338)	Overall (*N* = 824)
**Age**			
≤74	246 (50.6%)	172 (50.9%)	418 (50.7%)
75–87	190 (39.1%)	127 (37.6%)	317 (38.5%)
≥ 88	50 (10.3%)	39 (11.5%)	89 (10.8%)
**Sex**			
Male	140 (28.8%)	106 (31.4%)	246 (29.9%)
Female	346 (71.2%)	232 (68.6%)	578 (70.1%)
**Race**			
White	369 (75.9%)	252 (74.6%)	621 (75.4%)
Black	55 (11.3%)	36 (10.7%)	91 (11.0%)
Others	62 (12.8%)	50 (14.8%)	112 (13.6%)
**Tumor size**			
≤13	118 (24.3%)	86 (25.4%)	204 (24.8%)
14–44	284 (58.4%)	197 (58.3%)	481 (58.4%)
≥45	84 (17.3%)	55 (16.3%)	139 (16.9%)
**Grade**			
Well Differentiated	118 (24.3%)	83 (24.6%)	201 (24.4%)
Moderately Differentiated	235 (48.4%)	180 (53.3%)	415 (50.4%)
Poorly Differentiated	133 (27.4%)	75 (22.2%)	208 (25.2%)
**AJCC stage**			
I	130 (26.7%)	76 (22.5%)	206 (25.0%)
II	309 (63.6%)	223 (66.0%)	532 (64.6%)
IIIB	44 (9.1%)	39 (11.5%)	83 (10.1%)
IVB	3 (0.6%)	0 (0%)	3 (0.4%)
**T stage**			
T1a	46 (9.5%)	31 (9.2%)	77 (9.3%)
T1b	84 (17.3%)	51 (15.1%)	135 (16.4%)
T2	356 (73.3%)	256 (75.7%)	612 (74.3%)
**Regional nodes removed**			
0	267 (54.9%)	155 (45.9%)	422 (51.2%)
1–2	144 (29.6%)	105 (31.1%)	249 (30.2%)
≥3	75 (15.4%)	78 (23.1%)	153 (18.6%)
**Regional nodes positive**			
0	439 (90.3%)	299 (88.5%)	738 (89.6%)
1–3	44 (9.1%)	39 (11.5%)	83 (10.1%)
≥4	3 (0.6%)	0 (0%)	3 (0.4%)
**Surgery**			
Local tumor excision	4 (0.8%)	5 (1.5%)	9 (1.1%)
Simple/partial surgical removal of the primary site	117 (24.1%)	98 (29.0%)	215 (26.1%)
Total surgical removal of the primary site	341 (70.2%)	208 (61.5%)	549 (66.6%)
Surgery stated to be debulking	2 (0.4%)	0 (0%)	2 (0.2%)
Radical surgery	22 (4.5%)	27 (8.0%)	49 (5.9%)
**Survival status**			
Alive	197 (40.5%)	245 (72.5%)	442 (53.6%)
Dead	289 (59.5%)	93 (27.5%)	382 (46.4%)
**Cancer-specific death**			
Alive or dead of other cause	290 (59.7%)	269 (79.6%)	559 (67.8%)
Dead	196 (40.3%)	69 (20.4%)	265 (32.2%)
**Survival months**			
Mean (SD)	40.3 (32.7)	16.5 (13.9)	30.5 (29.1)
Median [min, max]	34.0 [0, 117]	13.0 [0, 47.0]	20.0 [0, 117]

In addition, 60 and 249 patients in the derivation cohort with T1b or T2 underwent total surgical removal of the primary site, and 5 and 14 underwent radical surgery; besides, 27 and 165 patients with T1b or T2 underwent total surgical removal of the primary site, and 3 and 24 patients underwent radical surgery in the validation cohort (Tables 2, 3). According to the cutoff points of regional lymph nodes removed, decided by X-tile (Figure 2), 267 (54.9%) and 155 (45.9%) patients had no regional lymph nodes removed, 144 (29.6%) and 105 (31.1%) had 1 or 2 regional lymph nodes evaluated, and 75 (15.4%) and 78 (23.1%) had more than or equal to 3 lymph nodes retrieved separately in derivation and validation groups (Table 1), respectively. Additionally, 157 and 17 patients who underwent total surgical removal of the primary site or radical surgery in the derivation cohort had at least one lymph node evaluated, while in the derivation cohort, 115 and 23 patients underwent total surgical removal of the primary site or radical surgery (Tables 2, 3).

**Table 2:** Clinical characteristics of patients who underwent specific surgeries in the derivation cohort.

	Local tumor excision (*N* = 4)	Simple/partial surgical removal of the primary site (*N* = 117)	Total surgical removal of the primary site (*N* = 341)	Surgery stated to be debulking (*N* = 2)	Radical surgery (*N* = 22)
**Tumor size**					
≤13	1 (25.0%)	22 (18.8%)	88 (25.8%)	0 (0%)	7 (31.8%)
14–44	2 (50.0%)	73 (62.4%)	199 (58.4%)	0 (0%)	10 (45.5%)
≥45	1 (25.0%)	22 (18.8%)	54 (15.8%)	2 (100%)	5 (22.7%)
**Grade**					
Well differentiated	1 (25.0%)	23 (19.7%)	87 (25.5%)	0 (0%)	7 (31.8%)
Moderately differentiated	2 (50.0%)	57 (48.7%)	163 (47.8%)	1 (50.0%)	12 (54.5%)
Poorly differentiated	1 (25.0%)	37 (31.6%)	91 (26.7%)	1 (50.0%)	3 (13.6%)
**AJCC stage**					
I	1 (25.0%)	28 (23.9%)	92 (27.0%)	1 (50.0%)	8 (36.4%)
II	3 (75.0%)	73 (62.4%)	222 (65.1%)	1 (50.0%)	10 (45.5%)
IIIB	0 (0%)	15 (12.8%)	26 (7.6%)	0 (0%)	3 (13.6%)
IVB	0 (0%)	1 (0.9%)	1 (0.3%)	0 (0%)	1 (4.5%)
**T stage**					
T1a	1 (25.0%)	10 (8.5%)	32 (9.4%)	0 (0%)	3 (13.6%)
T1b	0 (0%)	18 (15.4%)	60 (17.6%)	1 (50.0%)	5 (22.7%)
T2	3 (75.0%)	89 (76.1%)	249 (73.0%)	1 (50.0%)	14 (63.6%)
**Regional nodes removed**					
0	4 (100%)	72 (61.5%)	184 (54.0%)	2 (100%)	5 (22.7%)
1–2	0 (0%)	32 (27.4%)	109 (32.0%)	0 (0%)	3 (13.6%)
≥3	0 (0%)	13 (11.1%)	48 (14.1%)	0 (0%)	14 (63.6%)
**Regional nodes positive**					
0	4 (100%)	101 (86.3%)	314 (92.1%)	2 (100%)	18 (81.8%)
1–3	0 (0%)	15 (12.8%)	26 (7.6%)	0 (0%)	3 (13.6%)
≥4	0 (0%)	1 (0.9%)	1 (0.3%)	0 (0%)	1 (4.5%)

**Table 3:** Clinical characteristics of patients who underwent specific surgeries in the validation cohort.

	Local tumor excision (*N* = 5)	Simple/partial surgical removal of the primary site (*N* = 98)	Total surgical removal of the primary site (*N* = 208)	Radical surgery (*N* = 27)
**Tumor size**				
≤13	1 (20.0%)	25 (25.5%)	55 (26.4%)	5 (18.5%)
14–44	4 (80.0%)	58 (59.2%)	120 (57.7%)	15 (55.6%)
≥45	0 (0%)	15 (15.3%)	33 (15.9%)	7 (25.9%)
**Grade**				
Well differentiated	2 (40.0%)	25 (25.5%)	48 (23.1%)	8 (29.6%)
Moderately differentiated	2 (40.0%)	51 (52.0%)	114 (54.8%)	13 (48.1%)
Poorly differentiated	1 (20.0%)	22 (22.4%)	46 (22.1%)	6 (22.2%)
**AJCC stage**				
I	2 (40.0%)	31 (31.6%)	40 (19.2%)	3 (11.1%)
II	1 (20.0%)	59 (60.2%)	145 (69.7%)	18 (66.7%)
IIIB	2 (40.0%)	8 (8.2%)	23 (11.1%)	6 (22.2%)
**T stage**				
T1a	2 (40.0%)	13 (13.3%)	16 (7.7%)	0 (0%)
T1b	2 (40.0%)	19 (19.4%)	27 (13.0%)	3 (11.1%)
T2	1 (20.0%)	66 (67.3%)	165 (79.3%)	24 (88.9%)
**Regional nodes removed**				
0	2 (40.0%)	56 (57.1%)	93 (44.7%)	4 (14.8%)
1–2	1 (20.0%)	24 (24.5%)	72 (34.6%)	8 (29.6%)
≥3	2 (40.0%)	18 (18.4%)	43 (20.7%)	15 (55.6%)
**Regional nodes positive**				
0	3 (60.0%)	90 (91.8%)	185 (88.9%)	21 (77.8%)
1–3	2 (40.0%)	8 (8.2%)	23 (11.1%)	6 (22.2%)
≥4	0 (0%)	0 (0%)	0 (0%)	0 (0%)

### Selection of variables and establishment of the nomogram for OS and CSS

**Figure 3:**
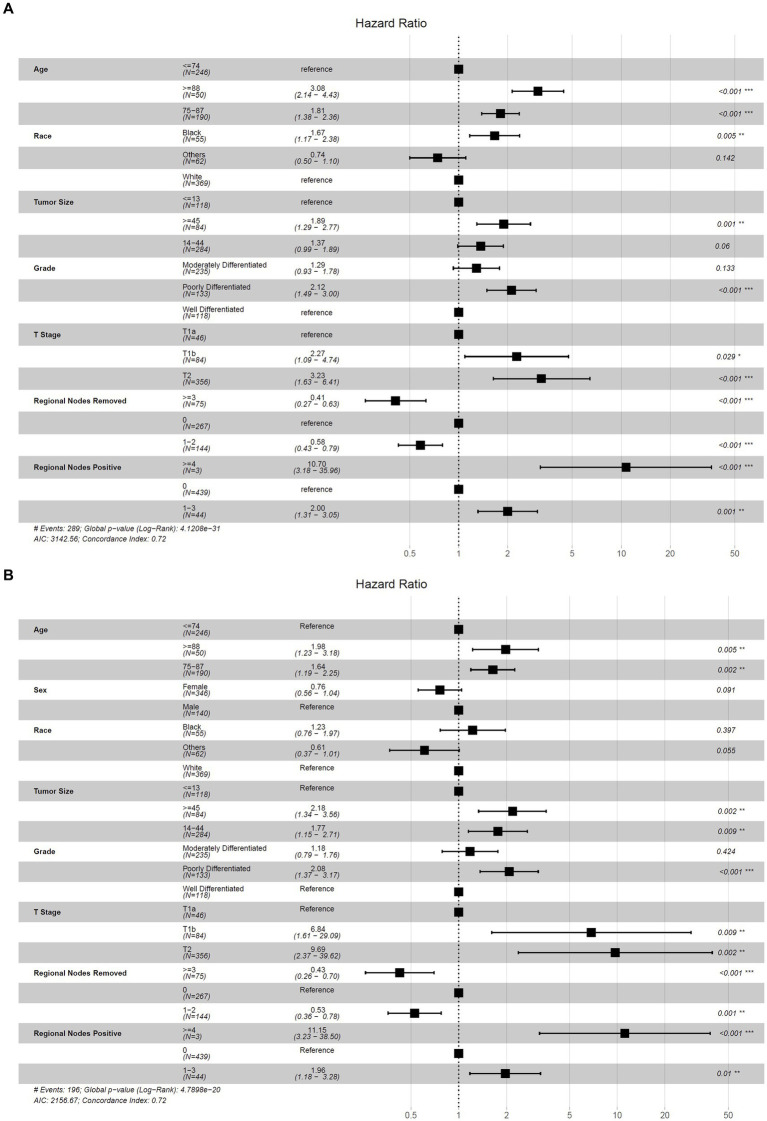
Forest plot for the hazard ratio of selected nomogram variables to predict OS and CSS. **(A)** Selected nomogram variables to predict OS; **(B)** selected nomogram variables to predict CSS.

The results obtained from stepwise selection analysis of the derivation cohort are shown in Figure 3, which indicated that younger patients had a better OS (age 75–87, ≥88 vs. ≤74, HR = 1.81, 3.08, *p* < 0.001) and CSS (age 75–87, ≥88 vs. ≤74, HR = 1.64, 1.98, *p* = 0.002, 0.005). Female patients had a lower risk of cancer-specific death (vs. male, HR = 0.76, *p* = 0.091). Black patients showed the lowest OS (race black, others vs. white, HR = 1.67, 0.74, *p* = 0.005, 0.142) and CSS (race black, others vs. white, HR = 1.23, 0.61, *p* = 0.397, 0.055). A larger tumor size represented a greater risk of overall death (tumor size 14–44, ≥45 vs. ≤13, HR = 1.37, 1.89, *p* = 0.06, 0.001) and cancer-specific death (tumor size 14–44, ≥45 vs. ≤13, HR = 1.77, 2.18, *p* = 0.009, 0.002). Well-differentiated patients had the highest OS (moderately-, poorly-, vs. well-differentiated, HR = 1.29, 2.12, *p* = 0.133, < 0.001) and CSS (moderately-, poorly-, vs. well-differentiated, HR = 1.18, 2.08, *p* = 0.424, < 0.001). Regarding the T stage, regional lymph nodes removed and positive regional lymph nodes, T1a, ≥3 regional lymph nodes removed and 0 positive regional lymph node were associated with the highest OS (T1b, T2 vs. T1a, HR = 2.27, 3.23, *p* = 0.029, <0.001; regional lymph nodes removed 1–2, ≥3 vs. 0, HR = 0.58, 0.41, *p* = 0.005, <0.001; regional nodes positive 1–3, ≥4 vs. 0, HR = 2.00, 10.70, *p* = 0.001, <0.001) and CSS (T1b, T2 vs. T1a, HR = 6.84, 9.69, *p* = 0.009, 0.002; regional lymph nodes removed 1–2, ≥3 vs. 0, HR = 0.53, 0.43, *p* = 0.001, <0.001; regional nodes positive 1–3, ≥4 vs. 0, HR = 1.96, 11.15, *p* = 0.010, <0.001).

**Figure 4:**
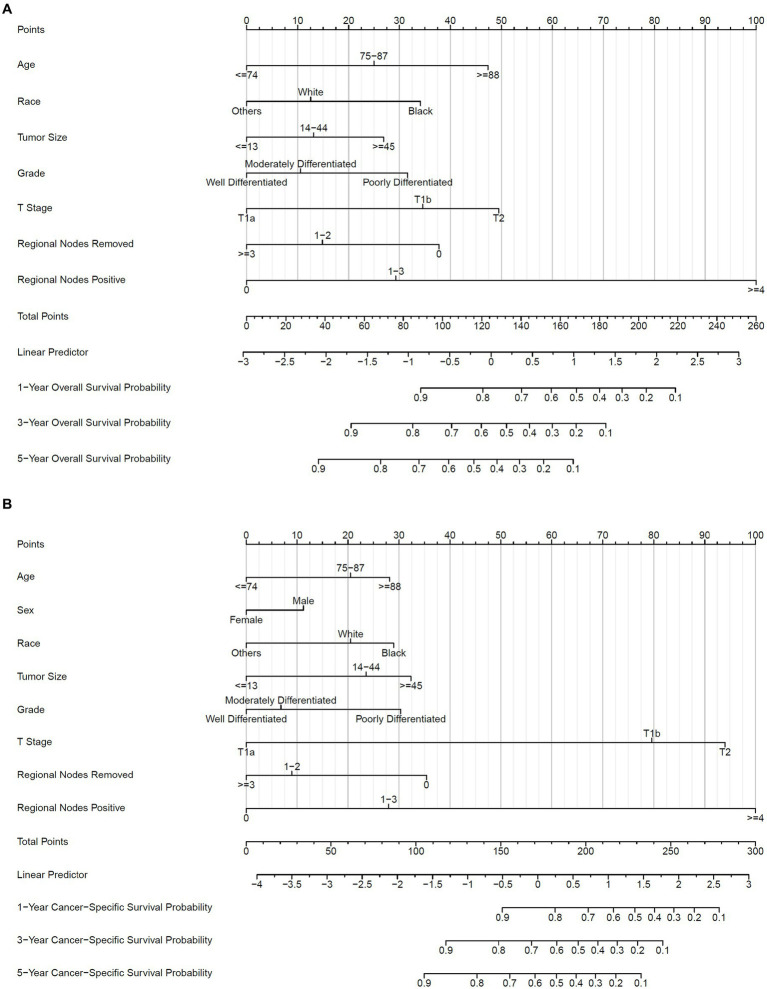
Nomogram to predict OS and CSS probability. **(A)** Prognostic nomogram of OS; **(B)** prognostic nomogram of CSS. 1-, 3-, and 5-year baseline OS probability: 0.9906716, 0.986361, 0.9809064; 1-, 3-, and 5-year baseline CSS probability: 0.997877, 0.9966245, 0.9946404.

Accordingly, these remaining variables were selected to establish the nomogram for 1-, 3-, and 5-year OS and CSS (Figure 4). As shown, ages 75–87 and ≥88 years old scored 25.0 and 47.4 in the OS nomogram and 20.5 and 28.2 in the CSS nomogram, respectively. White and black patients scored 12.6 and 34.1 in the OS nomogram and 20.5 and 29.0 in the CSS nomogram, respectively. The points of tumor size 14–44 and ≥45 mm were 13.1 and 26.9 in the OS nomogram, while 23.5 and 32.3 in the CSS nomogram, respectively. Grades moderately and poorly differentiated were 10.6 and 31.6 in the OS nomogram and 6.8 and 30.3 in the CSS nomogram, respectively. T1b and T2 were marked as 34.6 and 49.5 in the OS nomogram and 79.6 and 94.0 in the CSS nomogram, respectively. The regional lymph nodes removed 0 and 1–2 were assigned as 37.8 and 14.9 in the OS nomogram, and those in the CSS nomogram were 35.4 and 9.0, respectively. Finally, the scores of 1–3 and ≥4 positive regional lymph nodes were 29.3 and 100.0 in the OS nomogram, and 28.0 and 100.0 in the CSS nomogram, respectively. Remarkably, the male patients scored 11.3 in the CSS nomogram. By accumulating points of each variable, we could reveal the individual non-metastatic postoperative gallbladder adenocarcinoma patients’ probability of OS and CSS using the following formulas in Table 4.

**Table 4:** Formulas for calculating the OS and CSS probability corresponding to the total points.

Overall survival probability	
1-year	2.71e-07 × points^3–0.000150907 × points^2 + 0.019851086 × points + 0.1345431
3-year	2.71e-07 × points^3–0.000121908 × points^2 + 0.010127186 × points + 0.662659796
5-year	2.71e-07 × points^3–0.000108376 × points^2 + 0.006297085 × points + 0.798619656
**Cancer-specific survival probability**	
1-year	2.86e-07 × points^3–0.000210992 × points^2 + 0.043588063 × points-1.858700132
3-year	2.86e-07 × points^3–0.000182352 × points^2 + 0.030461372 × points-0.628424113
5-year	2.86e-07 × points^3–0.000171401 × points^2 + 0.025947168 × points-0.268809637

**Figure 5:**
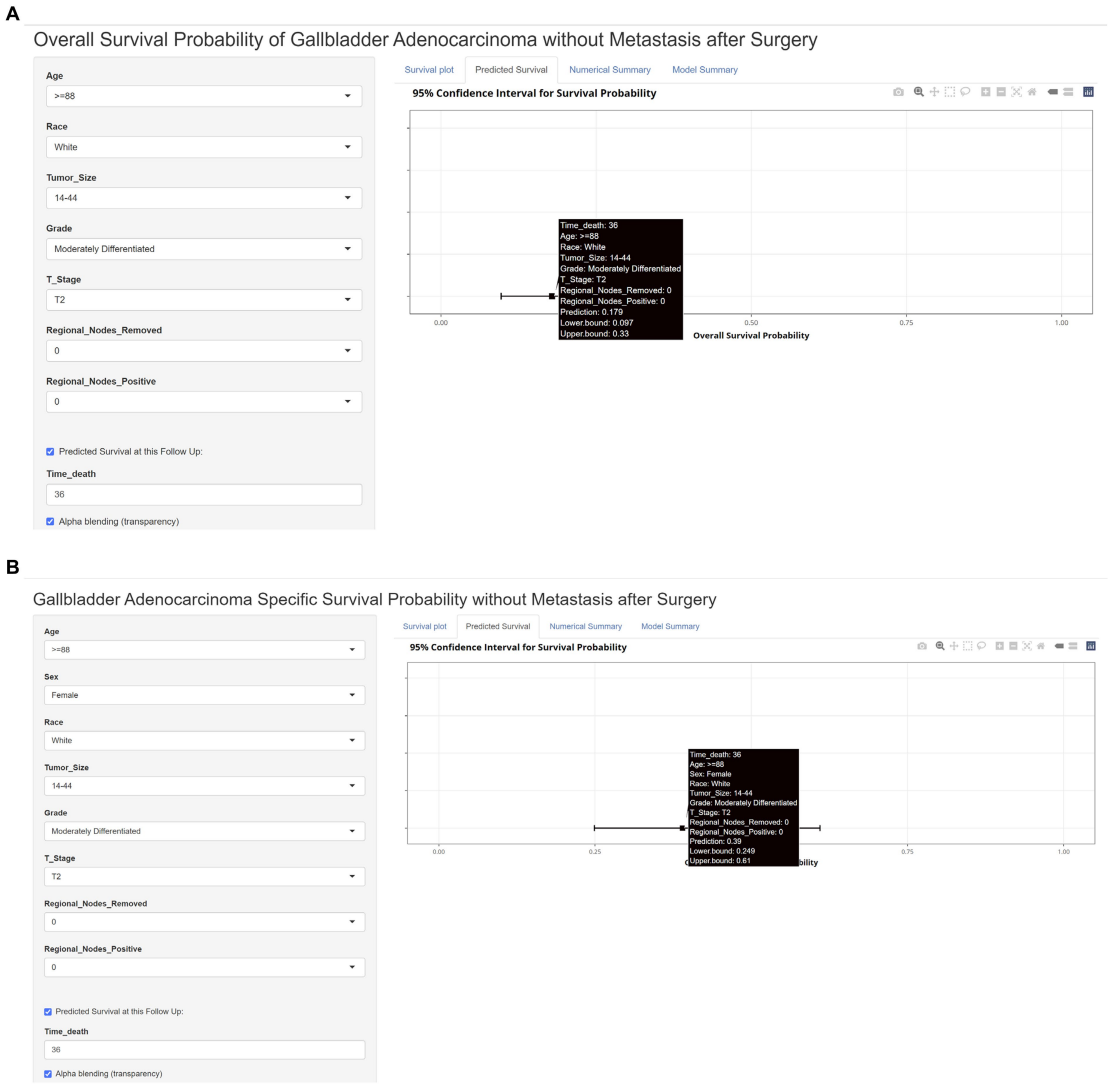
Web dynamic nomogram calculator with a clinical example. **(A)** Web dynamic nomogram calculator of OS with a clinical example; **(B)** web dynamic nomogram calculator of CSS with a clinical example.

Finally, we built a web calculator of the dynamic nomogram for clinicians to use conveniently online at https://jackycome.shinyapps.io/osgba_revised/ and https://jackycome.shinyapps.io/cssgba_revised/. For example, if a white female, aged greater than or equal to 88, with a tumor size between 14 and 44 mm, grade moderately differentiated, on T2 stage, 0 regional lymph nodes removed, and 0 positive regional lymph node, her 3-year OS and CSS probabilities were 0.179 and 0.390, respectively (Figure 5).

### Model performance and model validation

**Figure 6:**
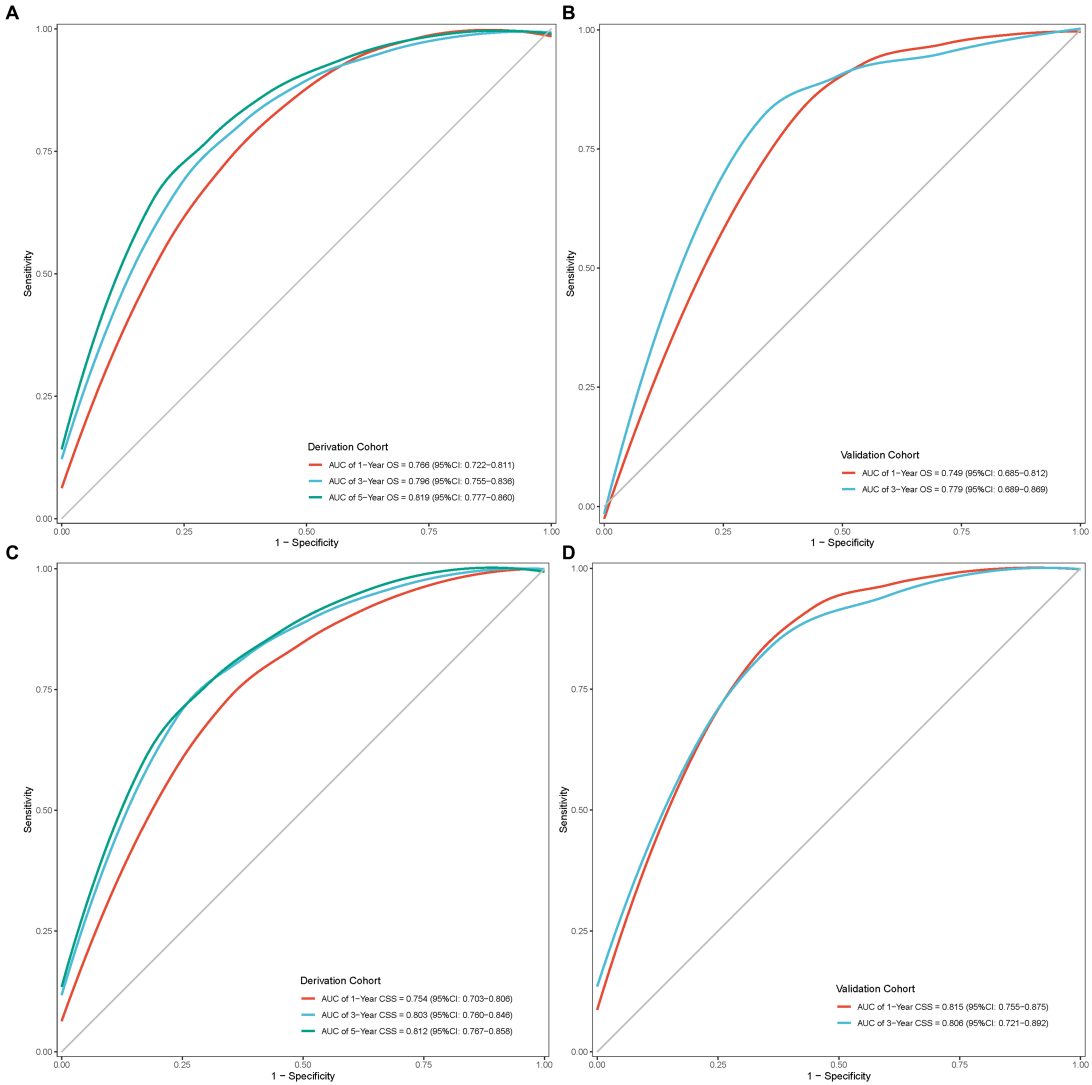
ROC curves of the nomogram model. **(A)** ROC curve of OS in the derivation cohort; **(B)** ROC curve of OS in the validation cohort; **(C)** ROC curve of CSS in the derivation cohort; **(D)** ROC curve of CSS in the validation cohort.

The internal and external assessment of nomogram performance was measured by Harrell’s C statistic and Brier score. Harrell’s C statistic of OS and CSS nomogram was 0.722 (95% confidence interval (95% CI): 0.693–0.750) and 0.721 (95% CI: 0.686–0.756) in the derivation cohort, while 0.701 (95% CI: 0.647–0.755) and 0.764 (95% CI: 0.712–0.817) in the validation cohort, respectively. Furthermore, the ROC curve demonstrated a moderate accuracy in predicting 1-, 3-, and 5-year OS and CSS in the derivation cohort, with an AUC of 0.766 (95% CI: 0.722–0.811), 0.796 (95% CI: 0.755–0.836), and 0.819 (95% CI: 0.777–0.860) in OS, and 0.754 (95% CI: 0.703–0.806), 0.803 (95% CI: 0.760–0.846), and 0.812 (95% CI: 0.767–0.858) in CSS, respectively (Figures 6A,C). Meanwhile, the ROC curve also showed good discrimination of 1-and 3-year OS and CSS prediction in the validation cohort, with an AUC of 0.749 (95% CI: 0.685–0.812) and 0.779 (95% CI: 0.689–0.869) in OS, and 0.815 (95% CI: 0.755–0.875) and 0.806 (95% CI: 0.721–0.892) in CSS, respectively (Figures 6B,D).

**Figure 7:**
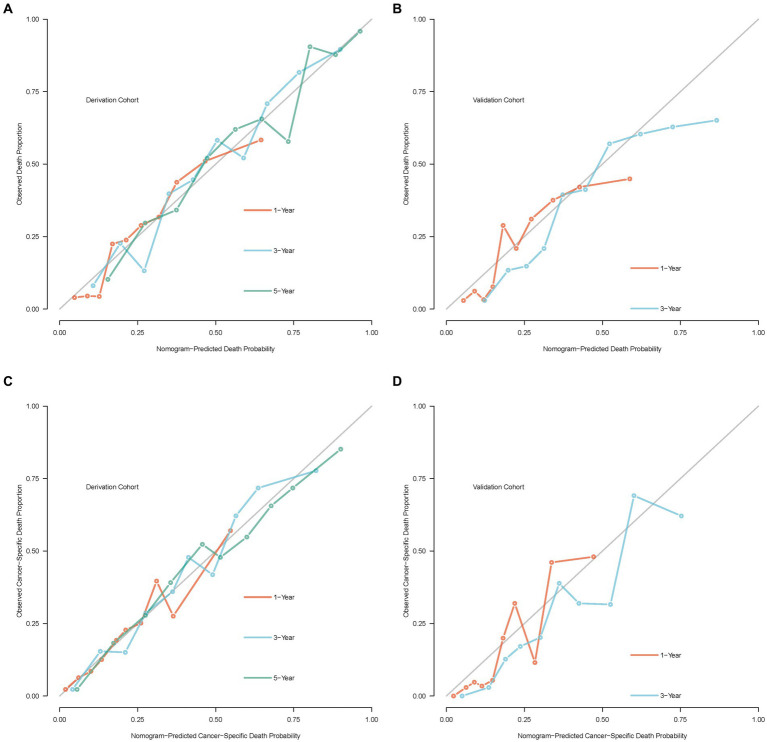
Calibration curves of the nomogram model. **(A)** Calibration curve of OS in the derivation cohort; **(B)** calibration curve of OS in the validation cohort; **(C)** calibration curve of CSS in the derivation cohort; **(D)** calibration curve of CSS in the validation cohort.

In addition, the calibration curve displayed perfect reliability between the death or cancer-specific death probability with observed death or cancer-specific death proportion in the derivation cohort, with the Brier scores of 1, 3, and 5 years being 0.165 (95% CI: 0.147–0.183), 0.185 (95% CI: 0.168–0.201), and 0.170 (95% CI: 0.151–0.188) in OS, and 0.145 (95% CI: 0.126–0.165), 0.175 (95% CI: 0.157–0.192), and 0.175(0.156–0.194) in CSS, respectively (Figures 7A,C). While in the validation cohort, the Brier scores of 1 and 3 years were 0.157 (95% CI: 0.132–0.181) and 0.191 (95% CI: 0.151–0.230) in OS, and 0.119 (95% CI: 0.097–0.142) and 0.160 (95% CI: 0.129–0.191) in CSS, respectively (Figures 7B,D).

## Discussion

As a common malignant biliary system tumor, the clinical manifestations of gallbladder cancer were obscure, most patients are clinically advanced with early metastasis, and the prognosis is unsatisfactory (1, 2). Therefore, high-quality research is urgently needed to break through the bottleneck of early diagnosis and following treatment. Along with the continuous development of medical science and technology, early diagnosis and radical surgical resection are still possible means to cure gallbladder cancer (22). In recent years, morbidity and mortality have shown a slow upward trend, with more than 90% adenocarcinoma (3–6). Age standard incidence rate of gallbladder cancer is 2.3 per 100,000 people on average globally, with the highest in East Asia and South America, with the incidence in men and young people increasing as well (6, 23, 24). Since the spring-up of laparoscopic surgery, gallbladder cancers have been spotted earlier in some patients, contributing to a higher chance of survival (25). Altiok et al. observed a phenomenal growth in the number of incidental gallbladder cancers after cholecystectomy operations over the past 20 years, of whom 90% were T1a, T1b, and T2, and 92.5% were adenocarcinoma (26). Furthermore, surgery is the only potential way for patients with gallbladder cancer to be cured and to survive for a long time (8, 27). Hence, it is very meaningful to predict the prognosis of incidental gallbladder adenocarcinoma patients diagnosed after open or laparoscopic cholecystectomy without distant metastasis.

The therapeutic regimen for incidental gallbladder adenocarcinoma differs from its stage. For stage T1a, simple cholecystectomy is adequate in over 90% of patients, and extended cholecystectomy, including lymphatic dissection, should be considered for T1b or more advanced stages (8). Wang et al. reported no positive lymph nodes observed in T1b gallbladder adenocarcinoma with tumor size <1 cm, indicating that simple cholecystectomy is curative for these patients, minimalizing the re-operation need (28). In particular, seven variables of age, race, tumor size, histologic grade, T stage, regional lymph nodes removed, and positive regional lymph nodes were finally determined to establish the OS prediction nomogram. Similarly, sex had also been added to the CSS nomogram, in which female was recognized as a protective factor. Additionally, the nomograms demonstrated a moderate accuracy of Harrell’s C statistic and induced a well-behaved calibration plot for OS and CSS prediction in both deviation and validation cohorts. Nevertheless, our nomograms overestimated 3-year overall death and cancer-specific death risks. This might be because the upper limit of follow-up in the temporal validation cohort is 47 months, and the median and mean follow-up time are 13.0 and 16.5 months, respectively, leading to excessive censored data. Due to short follow-up time, many cases have not yet observed death events until the last correspondence for the survival calculation. Other than that, overall and cancer-specific baseline survival probability is higher in the validation cohort. With the increasing medical technologies and living standards improvements, the average age is steadily rising globally, bringing about the elderly’s healthier and longer lives (29). Thus, we need to wait for the SEER database to be updated before re-evaluating and re-verifying our nomogram model.

The TNM staging system only considers the depth of tumor invasion but not the tumor size (19). Various researchers have found that tumor size could influence the prognosis of gallbladder cancer patients. However, the best cutoff points of tumor size to forecast the survival outcome remain arbitrary. Zhang et al. advocated that larger tumor size significantly contributed to the development of more advanced T stage, more frequent distant metastasis, and more positive regional lymph nodes, which was a confounding variable in predicting the CSS of postoperative gallbladder cancer patients (30). Zhang et al. developed a CSS model setting tumor size >3 cm as an essential prognostic predictor in gallbladder cancer patients without distant metastasis after surgical operation (18). In contrast, Yadav et al. created a clinically based predictive scoring nomogram for gallbladder cancer patients in which tumor size ≥5 cm and worse OS went hand in hand (31). Generally, increased gallbladder cancer mortality might be associated with increased age (1). However, a thorough investigation of the association between age and overall or gallbladder cancer-specific death risk remains unexplored without highlighting the predictive capacity of age until now. To facilitate clinical application, X-tile software that illustrates a graphical construction of a two-dimensional projection of every possible subpopulation was used to optimize outcome-based cut-point optimization of consecutive age and tumor size variables (20, 21). This study implied that age played a more crucial role in OS than the CSS nomogram, with ≥88 years old scoring 47.4 in OS but 28.2 points in CSS. Our study’s best cutoff values of tumor sizes were 14 and 44 mm, showing that larger tumor size was in line with a higher overall or gallbladder adenocarcinoma-specific death probability. Tumor sizes of 14–44 mm and ≥45 mm had 1.37 and 1.89 times higher overall death risk and 1.77 and 2.18 times higher gallbladder adenocarcinoma-specific death risk than tumor size ≤13 mm, respectively. Meanwhile, we found that a higher number of lymph nodes removed was a protective factor as none of the regional lymph nodes removed were associated with the lowest OS and CSS. In contrast to 0 regional lymph nodes removed, the hazard ratio of 1–2 and ≥3 regional lymph nodes removed were 0.58 and 0.41 in overall death risk and 0.53 and 0.43 in cancer-specific death risk, respectively. It suggested that at least one regional lymph node should be retrieved to ensure patients received adequate treatment. Given the above factors, our nomograms attributed that age, tumor size, and regional lymph nodes removed are essential determinants in OS or CSS prediction models for incidental gallbladder adenocarcinoma patients without distant metastasis who underwent surgery. Additionally, based on the current data, there was no significant correlation between the specific surgical methods and the prognosis, and radical surgery did not seem to benefit patients more, indicating that standard cholecystectomy is appropriate for these elderly patients with incidental gallbladder adenocarcinoma, minimalizing the second extended resection need.

Nonetheless, SEER, one of the largest tumor databases, was applied to develop and validate a prognostic nomogram for incidental gallbladder adenocarcinoma patients without distant metastasis after surgery; the drawback of this framework was its retrospective nature, which is associated with inevitable selection bias and information bias (32). Furthermore, the follow-up time of the temporal validation cohort is not long enough. In addition, due to the small retrospective cohort size of this study, more large-scale prospective studies or an updated SEER database are needed to re-validate our conclusions. The AUC of our developed nomogram was between 0.749 and 0.819 from the derivation and validation cohorts, which only offers a moderate accuracy in predicting prognosis for incidental gallbladder adenocarcinoma patients without distant metastasis after surgery. To counsel potential parents on prognosis prediction, clinicians should take complete account of these drawbacks when using these prognostic nomograms. According to the latest 8th version of the AJCC TNM classification system, the T2 category is separated into T2a and T2b based on tumor location on the gallbladder’s peritoneal or hepatic side. Our next step of updating the model by adding a subdivided T2 stage might improve the accuracy of the nomogram.

## Conclusion

Our study established novel dynamic nomograms based on seven and eight clinical variables separately to predict OS and CSS of incidental gallbladder adenocarcinoma patients without distant metastasis after surgery. The internal and external validation of the nomogram showed a moderate accuracy performance. Notwithstanding some limitations, these nomograms will assist doctors in advising and guiding therapeutic strategies for postoperative gallbladder adenocarcinoma patients conveniently. In the future, more randomized controlled trials are needed to update these nomograms.

## Data availability statement

The original contributions presented in the study are included in the article/supplementary material, further inquiries can be directed to the corresponding author.

## Ethics statement

The requirement of ethical approval was waived by the Ethics Committee of Hangzhou TCM Hospital Affiliated to Zhejiang Chinese Medical University for the studies on humans because the available data involved in our study was from public databases. The studies were conducted in accordance with the local legislation and institutional requirements. Written informed consent for participation was not required from the participants or the participants’ legal guardians/next of kin in accordance with the national legislation and institutional requirements. The human samples used in this study were acquired from the Surveillance, Epidemiology, and End Results (SEER) database.

## Author contributions

JC and YH conceived and designed the experiments. JC performed data analysis and drafted the manuscript. YH carried out the literature research and edited the manuscript. All authors contributed to the article and approved the submitted version.
